# Agar-reduced graphene oxide selectively adsorbs organic dyes and strengthens double-network hydrogels†

**DOI:** 10.1039/d0ra05735e

**Published:** 2020-08-07

**Authors:** Tang Tang, Karel Goossens, Sherilyn J. Lu, Dongli Meng, Christopher W. Bielawski

**Affiliations:** Center for Multidimensional Carbon Materials (CMCM), Institute for Basic Science (IBS) Ulsan 44919 Republic of Korea bielawski@unist.ac.kr; Department of Chemistry, Ulsan National Institute of Science and Technology (UNIST) Ulsan 44919 Republic of Korea; Department of Energy Engineering, Ulsan National Institute of Science and Technology (UNIST) Ulsan 44919 Republic of Korea

## Abstract

A straightforward and environmentally friendly method for synthesizing agar-reduced graphene oxide (ArGO) was devised. The topological features and emergent physical properties displayed by the novel carbon material were controlled by varying its water content. Dehydrated films of ArGO were found to be stable in water due to the π–π stacking interactions that formed between the aromatic components of its constituent sheets. In contrast, porous variants of ArGO afforded hydrogels that exhibited high swelling capacities. The intrinsic mechanical strength, elasticity and chemical stability of the hydrogels were further enhanced through adaption into double-network analogues. Such hydrogels, which were prepared using a facile and efficient one-pot methodology, exhibited a high fracture stress upon compression, and retained their shape in basic aqueous environments. These features can be expected to enable water purification and tissue engineering applications, among others.

## Abstract 2

Agar-reduced graphene oxide was conveniently synthesized from readily available precursors and found to selectively adsorb cationic organic dyes as well as to enhance the mechanical properties exhibited by various types of double-network hydrogels.

## Graphical abstract

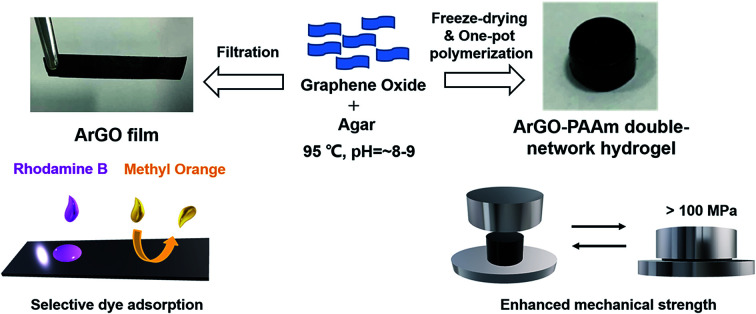

## Introduction

Reduced graphene oxide (rGO) is a relatively new material that has garnered wide interest for its attractive electronic and mechanical properties.^1–12^ The synthesis of rGO typically requires two steps. The first step consists of the oxidation of graphite using strong acids and oxidants (known as the Hummers method) in order to obtain graphene oxide (GO), which is then reduced in the second step.^13–17^ The reduction process partially recovers the electronic properties of graphene by restoring the sp^2^-hybridization of the constituent carbon atoms. Many methods have been used to reduce GO, including those that employ chemical reductants, light, or heat.^3,4,10,16^ Chemical reduction is often conducted with hydrazine,^18^ hydroquinone,^19^ dimethylhydrazine,^8^ or hydroiodic acid.^20^ Naturally-occurring substitutes, such as vitamin C (l-ascorbic acid),^21^ glucose,^22^ ethanol,^23^ proteins,^24^ green tea polyphenols,^25^ melatonin,^26^ wild carrot root,^27^ and starch^28^ have also been used to facilitate the reduction of GO, and often do so under mild conditions.

Hydrogels, which consist of cross-linked networks that are filled with water (generally 50–90 wt%), have found utility in a wide range of applications. For example, they have served as models for extracellular matrices, as actuators for optics and fluidic devices, as soft materials for the basis of engineered tissues, and as vehicles for drug delivery.^29–32^ Agar, which is a natural biopolymer composed of agarose, agaropectin and other polysaccharides, forms hydrogels upon combination with water. The sol–gel transitions of agar-based hydrogels have been attributed to random-coil-to-helix structural transitions that occur upon cooling from elevated temperatures and organize into bundles that form three-dimensional networks.^33^ Reduced GO-containing hydrogels have been synthesized by reducing GO using sodium ascorbate^34^ and employed in dye adsorption applications^35–38^ although selectivity remains a challenge.^39,40^

The preparation of hydrogels that combine high mechanical strength, toughness and elasticity represent an outstanding opportunity. While new polymerization methods for preparing strong yet flexible hydrogels have partially addressed the underlying issues and have afforded, for instance, double-network (DN) hydrogels,^41–45^ improved materials are needed to reduce the energy dissipated during loading–unloading cycles. GO and rGO have been shown to enhance the mechanical strength of various nanocomposite materials,^46–48^ including hydrogels, but the compression stress exhibited by such materials is often relatively low.^49^

**Scheme 1:**
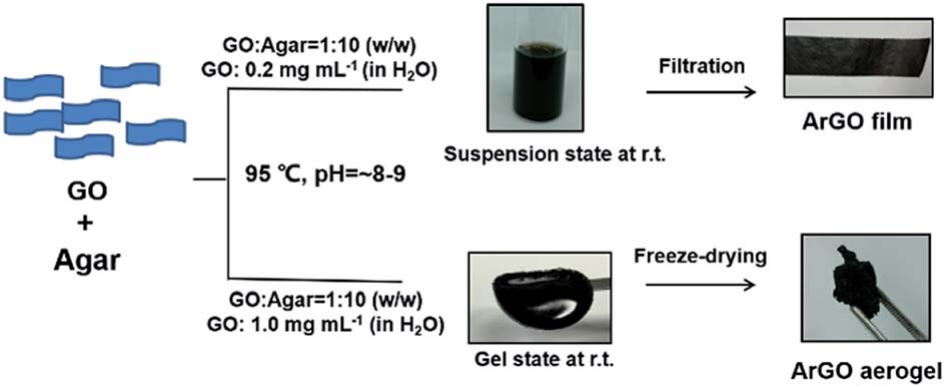
Summary of methodologies that were developed to synthesize and utilize ArGO.

We describe here a straightforward and environment-friendly method to synthesize and utilize a range of agar-reduced GO (ArGO) composites (Scheme 1). Based on previous reports of using naturally-occurring substitutes to reduce GO,^21,22,24–28^ agar, which is composed of hydroxyl-rich polysaccharides, was envisioned as a mild reducing agent. Moreover, the sol–gel phase transitions of aqueous mixtures of agar are thermoreversible: elevated temperatures afford solutions (85–120 °C) which turn into gels at lower temperatures (30–40 °C). It was envisioned that the reduction of GO would occur at elevated temperatures and the resulting rGO would be captured in the hydrogel that forms upon cooling. Indeed, as will be shown below, naturally occurring agar was used as a reducing agent to prepare rGO from GO under slightly basic conditions. Films comprised of ArGO were capable of selectively adsorbing dyes and significantly enhanced the mechanical strength and elastic properties of various types of hydrogels, primarily by increasing the water absorption capability. Collectively, these features should facilitate the use of ArGO in a broad range of contemporary applications, including water purification systems, engineered tissues and other components with biological relevance (*e.g.*, artificial skins), and as the basis of protective coatings.

## Results and discussion

### Reduction of GO using agar

GO was obtained *via* the oxidation of graphite using a modified Hummers method. Initial experiments explored using aqueous dispersions of GO (0.2 mg mL^−1^) to which agar powder was subsequent added (agar/GO = 10 : 1 w/w). To monitor the extent of the reduction reaction, UV-vis absorption spectra were separately recorded for an aqueous solution of agar powder (2.0 mg mL^−1^), an aqueous dispersion of GO (0.2 mg mL^−1^), and an aqueous mixture of agar (2.0 mg mL^−1^) and GO (0.2 mg mL^−1^) that was first heated to 95 °C for 12 h and then cooled to room temperature (Fig. S2a†). The maximum UV-vis signal recorded shifted from 226 nm for GO to 236 nm for the mixture of GO with agar, consistent with a reduction reaction.^28^ Although the reduction of GO was slow when only agar was used, the process was accelerated by adjusting the pH to approximately 8–9 using aqueous ammonia. Under alkaline conditions and in the absence of agar, the UV-vis peak maximum shifted to 240 nm within 3 h. When agar was included at the same pH, the UV-vis peak maximum shifted from 226 nm to 273 nm in the same amount of time (Fig. S2b†), and the absorption spectrum did not change thereafter (Fig. S2c†). During the reaction of GO with agar, the color of the mixture changed from brown to black (Fig. S2d†). The reaction product showed good dispersion characteristics in water and the aqueous dispersions were found to be stable, even after a period of six months.

**Fig. 1:**
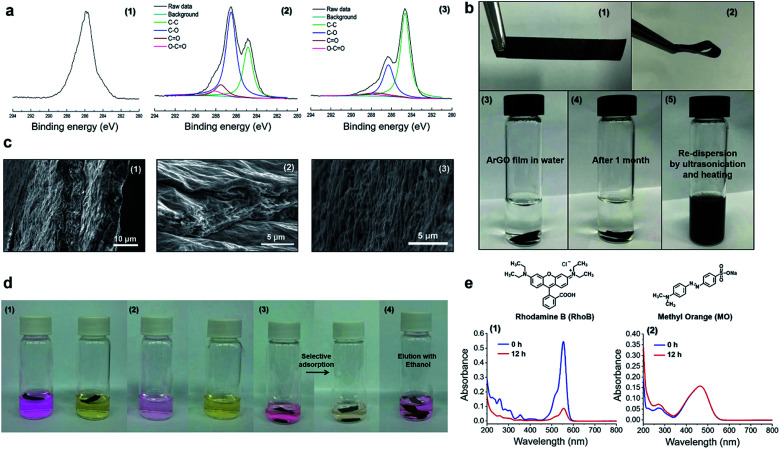
(a) C 1s XPS spectra that were recorded for (1) agar, (2) GO, and (3) ArGO. (b) Photographs of (1–2) an ArGO film; (3) an ArGO film in water; (4) an ArGO film in water after 1 month; (5) an ArGO film that underwent dispersion in water after being sonicated and heated to 95 °C. (c) SEM data recorded for the ArGO films including cross-section (1–2) and surface images (3). (d) Photographs of (1) ArGO films in aqueous solutions of RhoB (left) and MO (right) taken immediately after the ArGO films were added; (2) the same vials shown in the preceding panel after the ArGO films were soaked for 12 h in the solutions (the ArGO films were removed for clarity); (3) an ArGO film in an aqueous mixture of RhoB and MO taken immediately after the addition of ArGO (left) and after 12 h (right); and (4) a vial containing an ArGO film loaded with RhoB after soaking in ethanol for 1 h. (e) UV-vis absorption spectra recorded for (1) an aqueous solution of RhoB immediately after the addition of ArGO (blue) and after 12 h (red); (2) an aqueous solution of MO immediately after the addition of ArGO (blue) and after 12 h (red).

The isolated reaction product was further investigated using X-ray photoelectron spectroscopy (XPS).^18^ A broad C 1s XPS signal with a maximum at 286.4 eV was observed and attributed to the C–OH and C–O–C groups in agar (*c.f.*, Fig. 1a(1 and 3)). For comparison, XPS data that were recorded for the GO starting material showed four C 1s signals that were centered at 284.6, 286.8, 287.7 and 288.1 eV, and assigned to aromatic C=C/C–C, C–O, C=O and (C=O)–O bonds, respectively (Fig. 1a(2)). Moreover, the C 1s signals originating from oxygenated carbon atoms had significantly decreased upon treatment of GO with agar, consistent with a reduction reaction. The signal centered at 286.4 eV may stem from residual agar moieties attached to the surface of the ArGO product.

Next, a type of “ArGO paper” was prepared using a vacuum filtration technique. It has been previously shown that GO sheets can assemble into highly ordered structures under the directional flow that is induced by vacuum filtration.^50^ Such GO paper is not stable in water and slowly re-disperses, a process that is accompanied by a change in color of the water layer to brown (Fig. S3†). In contrast, ArGO paper, which was prepared using an analogous method, was found to mechanically robust and stable in water (Fig. 1b). Although the film did not expand or disintegrate over the course of one month after being immersed in water, it could be re-dispersed by ultrasonication (30 min) followed by heating to 95 °C (10 min). The unique characteristics were attributed to the interplay of the π–π stacking interactions between the aromatic fragments in the ArGO paper which maintain integrity in aqueous media and the presence of agar fragments on the material's surface which facilitate dispersion in water. Indeed, stacked ArGO platelets can be observed in the cross-section scanning electron microscopy (SEM) images recorded for an ArGO film (Fig. 1c).

### Selective adsorption properties displayed by ArGO films

SEM images of ArGO films revealed the presence of rough surfaces with many pits and holes (Fig. 1c). The observations prompted a test of the material's adsorption capabilities using rhodamine B (RhoB) and methyl orange (MO), which are common organic dyes, as model compounds. Pieces of ArGO paper (1 mg) were independently soaked in aqueous solutions of the dyes ([dye]_0_ = 2 mg L^−1^, 4 mL total volume) for 12 h. While the RhoB solution was initially pink, the color visually decreased over time due to adsorption of the dye onto the ArGO paper (Fig. 1d). In contrast, the initial yellow-orange color of the MO solution remained unchanged (Fig. 1d). The selective adsorption of RhoB *versus* MO was further demonstrated by soaking ArGO paper (1 mg) in an aqueous mixture of both dyes ([RhoB]_0_ = [MO]_0_ = 1 mg L^−1^, 2 mL total volume). Again, the pink color of the RhoB disappeared over time while the yellow-orange color of the dissolved MO persisted. The observations were quantified using UV-vis absorption spectroscopy (Fig. 1e and S4†). The signal intensity assigned to RhoB attenuated by 90% after 12 h whereas the signal intensity assigned to MO remained relatively constant. Adsorbed RhoB was later recovered by soaking the loaded ArGO paper in ethanol for 1 h. The selectivity may stem from charge complementarity as the cationic (ammonium) groups in RhoB should exhibit a stronger attraction to the anionic (carboxylate) groups present in the ArGO,^35,51^ particularly when compared to the anionic (sulfonate) groups found in MO. The RhoB adsorption capacity of the ArGO film was measured to be 39.6 mg g^−1^.

### Synthesis and study of ArGO-based hydrogels and aerogels

**Fig. 2:**
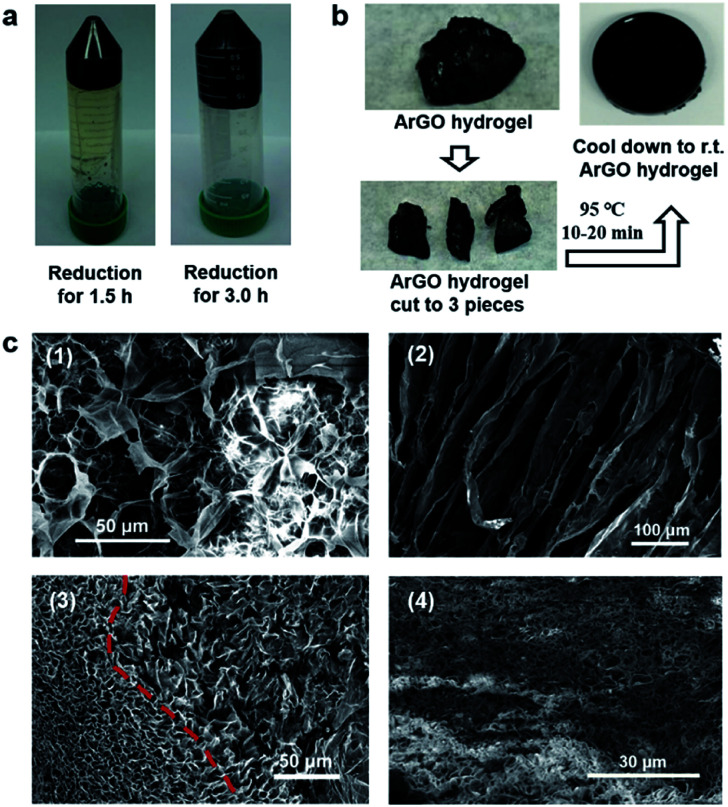
(a) Photographs of ArGO hydrogels that were obtained after centrifugation (4000 rpm), following GO reduction for 1.5 h (left) or 3 h (right). (b) Photographs of a re-shaping experiment (see text). (c) (1) SEM image of an agar aerogel (the SEM sample was coated with gold). (2) SEM image of an ArGO aerogel that was obtained from 1.0 mg mL^−1^ GO and agar/GO = 10 : 1 w/w (the SEM sample was not coated with gold). (3) SEM image of an ArGO aerogel that was obtained from 1.0 mg mL^−1^ GO and agar/GO = 5 : 1 w/w. (4) SEM image of an ArGO–PAAm DN aerogel (reduction conditions: agar/GO = 10 : 1 w/w; DN gel synthesis: 30 mg ArGO, 270 mg AAm, 0.1 mol% cross-linker MBAA relative to AAm, 1 mol% radical initiator APS relative to AAm, 1.5 mL water) (the SEM sample was coated with gold prior to analysis).

It was discovered that a black hydrogel (see Fig. S6a,† inset) was formed upon increasing the initial concentrations of GO and agar used in the aforementioned reduction reactions to 1.0 mg mL^−1^ (from 0.2 mg mL^−1^) while keeping the other conditions constant (*i.e.*, agar/GO = 10 : 1 w/w, pH = 8–9 using aqueous NH_3_, 95 °C). Even though a hydrogel had formed, the GO still underwent reduction as determined by UV-vis spectroscopy, which revealed that the hydrogel product absorbed at slightly longer wavelengths when compared to the starting material. Close inspection of the UV-vis data indicated that the reduction reaction reached completion within 3 h, a result that was confirmed through visual inspection as prematurely terminating the reaction after shorter periods of time (1.5 h) resulted in gels that were light brown in color and phase separated from the hydrogel upon centrifugation (Fig. 2a). The hydrogels formed aqueous solutions/dispersions upon heating to 85–120 °C which returned to a gel state upon cooling. The reversible feature enabled pieces of hydrogel to be combined into a larger piece through heating and subsequent cooling to room temperature (Fig. 2b).

The aforementioned hydrogels were freeze-dried, and the morphologies and microstructures of the resulting aerogels were studied by SEM. As shown in Fig. 2c, an ArGO-based aerogel (Fig. 2c(2)) displayed a more pronounced porosity than an aerogel that was obtained from agar (Fig. 2c(1), *i.e.*, in the absence of GO). The higher porosity may rationalize the relatively high water-swelling properties of ArGO. For example, a series of comparative water-swelling tests indicated that the ArGO-based hydrogels displayed up to a 100-fold higher water-swelling capability than hydrogels based on agar. In other words, 30 mg of ArGO was found to be capable of absorbing up to 3000 mg of water (Fig. S5†). Moreover, when an initial agar to GO ratio lower than 10 : 1 was used, the resulting products did not exhibit a clear sol–gel transition (Fig. S6†) and SEM of the corresponding ArGO aerogels revealed relatively small and broadly distributed pore sizes (*e.g.*, see Fig. 2c(3)). The observation was tentatively ascribed to the relatively large quantity of rGO present which underwent aggregation due to π–π stacking interactions and, in turn, inhibited the dispersion of ArGO in water.

### ArGO–PAAm double-network (DN) hydrogels

**Fig. 3:**
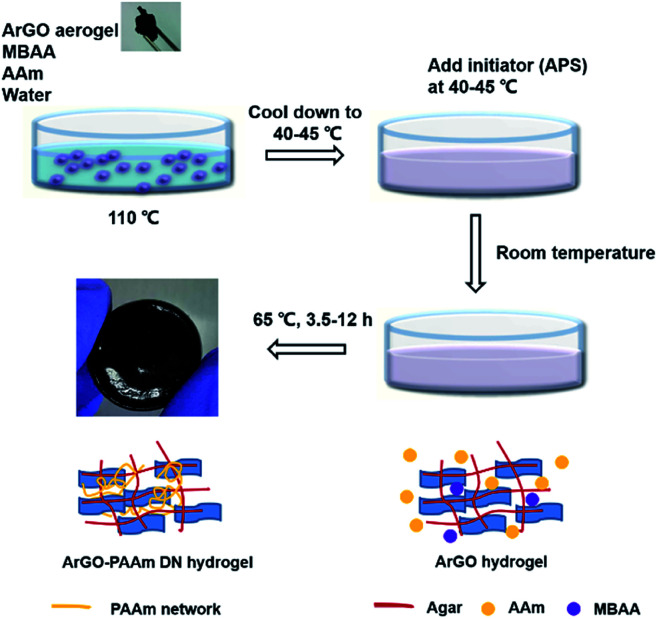
Procedure used to prepare ArGO–PAAm DN hydrogels and schematic representations of the gel structures at each stage.

Building on the aforementioned results, a series of DN hydrogels containing ArGO were prepared. While DN hydrogels are usually synthesized *via* multi-step, sequential free radical polymerization processes,^42,44^ a one-pot alternative method was envisioned.^41^ As summarized in Fig. 3, an ArGO aerogel was first prepared as described above. The precursors for the second network (*i.e.*, the AAm monomer and the MBAA cross-linker) and ArGO aerogel were mixed with water at 110 °C, a temperature that was above the sol–gel transition, to obtain a homogeneous black aqueous solution. After slowly cooling the solution to 40–45 °C, a temperature at which the mixture was still in a sol state, an aqueous solution of a radical initiator (APS) was added and then the temperature was decreased to room temperature to capture the monomers in the first network. The mixture was then reheated to 65 °C (note: the material was still in a gel state at this temperature) to induce polymerization of AAm and MBAA and thus formation of the second network.

The formation of the two networks was confirmed using SEM and various spectroscopic techniques. For example, SEM of an aerogel obtained upon dehydration of an ArGO–PAAm hydrogel exhibited relatively small pore sizes (Fig. 2c(4)), particularly when compared to ArGO aerogels described above. In addition, as shown in Fig. S9,† the FT-IR spectrum recorded for an ArGO–PAAm DN aerogel indicated that the second, PAAm-based network had formed as diagnostic signals assigned to primary amides (3340–3332 cm^−1^ and 1620–1640 cm^−1^), secondary amides (3250–3350 cm^−1^), N–H groups (3160–3190 cm^−1^) and C=O groups (1593–1603 cm^−1^) were observed. Concomitantly, signals assigned to the olefinic groups (960 cm^−1^ and 822 cm^−1^) of the acrylamide monomer disappeared.

**Fig. 4:**
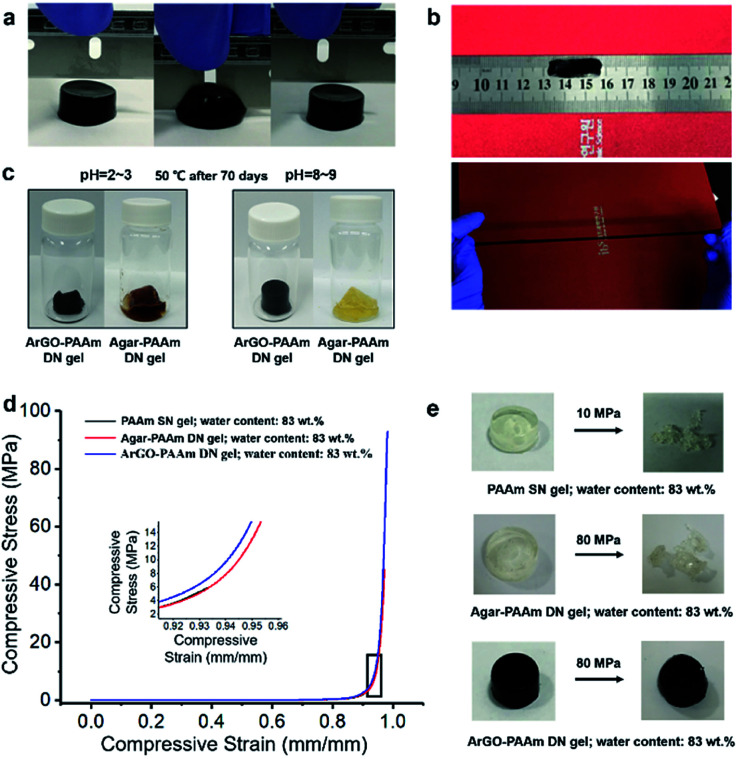
(a) ArGO–PAAm DN hydrogels exhibited no fracture after being cut by a blade (DN gel synthesis conditions: ArGO/AAm = 1 : 9 w/w; 0.1 mol% MBAA relative to AAm and 1 mol% APS relative to AAm; polymerization for 7 h at 65 °C). (b) ArGO–PAAm DN hydrogels exhibited an outstanding stretching capability (DN gel synthesis conditions: ArGO/AAm = 1 : 9 w/w; 0.06 mol% MBAA relative to AAm and 1 mol% APS relative to AAm; polymerization for 7 h at 65 °C). (c) In contrast to the agar–PAAm DN hydrogels, the integrity of the ArGO–PAAm DN hydrogels were preserved under alkaline conditions (pH = 8–9) (DN gel syntheses: agar/AAm = 1 : 9 w/w or ArGO/AAm = 1 : 9 w/w; 1 mol% MBAA relative to AAm and 1 mol% APS relative to AAm; polymerization for 7 h at 65 °C). (d) Summary of mechanical properties measured for different types of hydrogels (indicated). Representative stress–strain curves at 90% strain. (e) Photographs of different types of hydrogels under stress (indicated).

The mechanical properties and chemical reactivity of ArGO–PAAm DN hydrogels were first assessed in a qualitative manner. As shown in Fig. 4b, the gels were determined to be mechanically robust and could withstand significant elongation without breaking. To probe chemical stability, ArGO–PAAm DN hydrogels and agar–PAAm DN hydrogels, which were prepared as controls, were independently subjected to acidic and, separately, basic environments for 70 days at 50 °C (dilute HCl, pH = 2–3, or ammonium hydroxide, pH = 8–9, respectively). Although the gels lost their structural integrity under acidic conditions, the ArGO–PAAm DN gel was found to be relatively resistant to base (Fig. 4c).

The mechanical properties of the DN hydrogels were also quantitatively measured using a universal testing machine. To facilitate comparisons, PAAm single-network (SN) hydrogels and agar–PAAm DN hydrogels were also prepared and tested. Key results are shown in Fig. 4d, 5a and b. The compressive stress at 90% strain measured for the ArGO–PAAm DN hydrogel (average stress: 4.1 MPa) was found to be higher than that of the PAAm SN hydrogel (average stress: 1.8 MPa) and the agar–PAAm DN hydrogel (average stress: 2.5 MPa). Likewise, the fracture stress of the PAAm SN gel was measured to be low (average fracture stress: 4.8 MPa) with a fracture strain (*λ*) of 92%, whereas the ArGO–PAAm DN gel exhibited a significantly higher fracture stress (average fracture stress: 97.7 MPa, *λ* = 98%) than the agar–PAAm DN gel (average fracture stress: 48.0 MPa, *λ* = 97%). The fracture stress of the ArGO–PAAm DN gel is higher than that reported for conventional DN gels (90% water) made from poly(2-acrylamido-2-methylpropanesulfonic acid)–poly(acrylamide) (17.2 MPa and *λ* of 92%).^42^ The gel states observed before and after compression are shown in Fig. 4e. After being compressed at a pressure of 10 MPa, the structural integrity of the PAAm SN gel was compromised. The agar–PAAm DN gel was also compromised although a pressure of 80 MPa was required. In contrast, the ArGO–PAAm DN gel quickly recovered to its initial shape even after being compressed at high pressures (80 MPa).

**Fig. 5:**
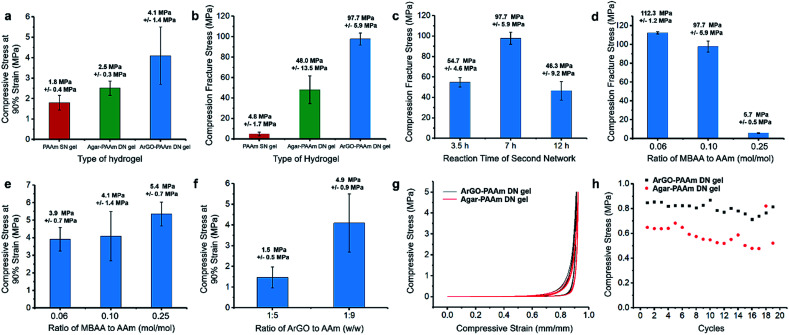
Mechanical properties of different types of hydrogels. (a) Compressive stress at 90% strain. (b) Fracture stress for different types of hydrogels (indicated). The error bars correspond to repeated measurements. Gel synthesis conditions: PAAm SN gel: 0.1 mol% MBAA relative to AAm and 1 mol% APS relative to AAm; water content: 83 wt%; polymerization for 7 h at 65 °C; agar–PAAm DN gel: agar/AAm = 1 : 9 w/w; 0.1 mol% MBAA relative to AAm and 1 mol% APS relative to AAm; water content: 83 wt%; polymerization for 7 h at 65 °C; ArGO–PAAm DN gel: ArGO/AAm = 1 : 9 w/w; 0.1 mol% MBAA relative to AAm and 1 mol% APS relative to AAm; water content: 83 wt%; polymerization for 7 h at 65 °C. (c) Influence of the second network polymerization time on the fracture stress of the resulting DN gels (DN gel synthesis conditions: ArGO/AAm = 1 : 9 w/w; 0.1 mol% MBAA relative to AAm and 1 mol% APS relative to AAm; water content: 83 wt%). Influence of the amount of MBAA cross-linker on (d) the fracture stress and (e) the compressive stress at 90% strain of the resulting DN gels (DN gel synthesis conditions: ArGO/AAm = 1 : 9 w/w; 1 mol% APS relative to AAm; water content: 83 wt%; polymerization for 7 h at 65 °C). (f) Influence of the weight ratio of ArGO to AAm on the compressive stress at 90% strain of the resulting DN gels (DN gel synthesis conditions: 0.1 mol% MBAA relative to AAm and 1 mol% APS relative to AAm; water content: 83 wt% for ArGO/AAm = 1 : 9 w/w and 89 wt% for ArGO/AAm = 1 : 5 w/w; polymerization for 7 h at 65 °C). Stress–strain curves (g) and loading–unloading cycling tests (h) for different types of DN hydrogels (stress: 5 MPa). Gel synthesis conditions: ArGO–PAAm DN hydrogel: ArGO/AAm = 1 : 9 w/w; 0.1 mol% MBAA relative to AAm and 1 mol% APS relative to AAm; water content: 83 wt%; polymerization for 7 h at 65 °C; agar–PAAm DN hydrogel: agar/AAm = 1 : 9 w/w; 0.1 mol% MBAA relative to AAm and 1 mol% APS relative to AAm; water content: 83 wt%; polymerization for 7 h at 65 °C. The error bars reflect repeated measurements.

To ascertain the origin of the beneficial effects provided by the second network in the aforementioned hydrogels, different polymerization conditions were explored. First, the polymerization time used to form the second network was varied between 3.5 h and 12 h. As shown in Fig. 5c, the DN gel obtained after 7 h of polymerization time featured a higher fracture stress when compared to those that were obtained over shorter (*i.e.*, 3.5 h) or longer (*i.e.*, 12 h) time periods. Presumably, the formation of the second network was not finished after 3.5 h, whereas the polymerization of AAm and MBAA had advanced to such an extent that the second network effectively disrupted the first network after 12 h. Next, different amounts of MBAA cross-linker were added (*i.e.*, 0.06, 0.1 and 0.25 mol% relative to AAm) while keeping the polymerization time at 7 h. Increasing the cross-linker content to 0.25 mol% led to a relatively high compressive stress at 90% strain but a low fracture stress (Fig. 5d and e). The results suggested to us that a higher cross-linker content increased the hardness of the DN gel but not its toughness. When a lower cross-linker content was used (*i.e.*, 0.06 mol%), the DN gels featured a lower hardness but were softer and thus could better manage the higher compressive stress (average fracture stress: 112.3 MPa, Fig. 5d). During the course of these studies, it was discovered that changing the weight ratio of the two networks from 1 : 9 to 1 : 5 (ArGO : PAAm) caused the water content of the gels to increase slightly from 83 wt% to 89 wt% and resulted in a lowered compressive stress at 90% strain (Fig. 5f), which may be due to the relatively higher water content in the latter. Finally, the recovery properties of ArGO–PAAm DN hydrogels and agar–PAAm DN hydrogels were compared through a series of loading–unloading cycling tests using a compression stress of 5 MPa. After the first cycle, the ArGO–PAAm DN gel recovered up to 80% of its initial strain, while the recovery of the agar-based DN gel was only about 60% (Fig. 5g and h). Even though the compressive strains of both types of gels gradually decreased over 20 cycles, the ArGO–PAAm DN gel still outperformed the agar–PAAm DN gel in terms of recovery properties.

## Conclusions

A facile and environment-friendly method that employs agar, a naturally occurring biopolymer, was developed to produce ArGO films and ArGO-based hydrogels. The ArGO films were stable in aqueous media yet could be re-dispersed in water using ultrasonication and subsequently re-processed. The films also exhibited good stability in water and were used to selectively adsorb cationic dyes in aqueous media. Hydrogels based on ArGO, including ArGO–PAAm DN hydrogels, exhibited high mechanical strength and elasticity. The DN hydrogels showed high fracture stress upon compression and retained their shape in basic aqueous environments. Moreover, the DN hydrogels were prepared using a facile one-pot methodology that should enable access to relatively complex shapes if appropriate molds are used. In a broader perspective, the beneficial features offered by ArGO can be expected to facilitate a wide range of water purification, tissue engineering, high-performance coatings, and other contemporary applications.

## Experimental section

### Materials

Graphene oxide (GO) was synthesized from graphite powder (SP-1, Bay Carbon, Inc.) utilizing a modified Hummers method.^15,50,52^ Agar powder and 35% aqueous ammonia were purchased from Alfa Aesar. Acrylamide (AAm), *N*,*N*′-methylenebis(acrylamide) (MBAA), ammonium persulfate (APS) and rhodamine B and methyl orange were purchased from Sigma-Aldrich.

### Characterization

Scanning electron microscopy (SEM) was conducted using a FEI Verios 460 High Resolution Field Emission instrument with an accelerating voltage of 5 kV and a working distance of 10 mm. Atomic force microscopy (AFM) images were recorded in the tapping mode using a Bruker Dimension Icon instrument. Prior to analysis, an aqueous dispersion of GO platelets was drop-casted onto a silicon wafer and allowed to dry in air. The AFM images were analyzed using the Gwyddion SPM software package. X-ray photoelectron spectroscopy (XPS) data were recorded on a Thermo Fisher Scientific ESCALAB 250Xi X-ray photoelectron spectrometer. Attenuated total reflectance (ATR) infrared (IR) spectra were recorded on an Agilent Cary-630 FT-IR spectrometer using a germanium crystal. Raman spectra were acquired with a Horiba LabRAM HR Evolution Raman microscope using a laser excitation wavelength of 532 nm. UV-vis absorption spectra were recorded on an Agilent Cary 100 UV-vis spectrometer outfitted with a Peltier multicell temperature controller. Compression tests were conducted on an Instron 5567 at a compression rate of 2 mm min^−1^ using cylindrical gel samples with a height of ∼10 mm and a diameter of ∼15 mm. The compressive strain was estimated as *h*/*h*_0_, where *h* is the height after deformation and *h*_0_ is the original height. The compressive stress was measured as *F*/*A*_0_, where *F* is the force applied on the gel and *A*_0_ is the original cross-sectional area.

### Preparation of ArGO films

In a typical procedure, 400 mg of agar was added to 200 mL of a 0.2 mg mL^−1^ aqueous suspension of GO. Aqueous ammonia (35%, 0.60 mL) was then added to adjust the pH to approximately 8–9. The reaction mixture was then heated to 95 °C for 3 h to facilitate the reduction of GO. After cooling to room temperature, the resulting dispersion was subjected to ultrasonication followed by centrifugation (15 000 rpm) and then decanted. Fresh water was added to the decanted portion, and then the resulting mixture was subjected to ultrasonication and centrifugation again. The entire procedure was repeated twice. A film was prepared by slow vacuum filtration of the washed ArGO aqueous dispersion (∼0.1 mg mL^−1^) on a hydrophilic PTFE nitrate membrane filter (47 mm diameter, 0.2 μm pore size). The resulting film was dried for 48 h at 120 °C in an oven under an atmosphere of air and then carefully peeled from the filter. The dehydrated ArGO paper could be re-dispersed in water by ultrasonication for 30 min followed by heating to 95 °C for 10 min.

### Dye adsorption

A piece of dehydrated ArGO film (1 mg) was added to an aqueous solution (4 mL) of rhodamine B (RhoB) and/or methyl orange (MO) (2 mg L^−1^). At regular intervals, the ArGO film was removed and a UV-vis spectrum of the dye solution was recorded. To test the adsorption capacity of a typical ArGO film, the film (1 mg) was added to an aqueous solution (2 mL) containing RhoB (20 mg L^−1^). At regular intervals, UV-vis spectra were recorded and photographs were taken. The amount of adsorbed dye (*q*) was calculated according to the following equation: *q* = [(*C*_0_ − *C*_*t*_)*V*]/*m*, where *C*_0_ and *C*_*t*_ are the initial dye concentration and the dye concentration at time *t*, respectively; *V* is the volume of the solution; and *m* is the mass of the film.

### Preparation of ArGO aerogels and ArGO–PAAm DN hydrogels

In a typical procedure, 600 mg of agar was added to 60 mL of a 1.0 mg mL^−1^ aqueous suspension of GO. Aqueous ammonia (35%, 0.40 mL) was then added to adjust the pH to approximately 8–9. The mixture was heated to 95 °C for 3 h to facilitate the reduction of GO. After cooling to room temperature, the mixture turned to a gel. The resulting hydrogel was subjected to ultrasonication followed by centrifugation (15 000 rpm) and then decanted. Fresh water was added to the decanted portion, and then the resulting mixture was subjected to ultrasonication and centrifugation again. The entire procedure was repeated twice. The washed ArGO hydrogel was then freeze-dried at −108 °C for 3 days to afford an ArGO-based aerogel. Next, a mixture of AAm (3.80 mmol, 0.270 g), MBAA cross-linker (3.80 μmol, 0.59 mg, 0.1 mol% relative to AAm), degassed water (1.3 mL) and ArGO aerogel (0.030 g) was mixed, heated to 110 °C for 10–15 min, and then cooled to 40–45 °C, at which temperature the mixture was in a sol state. Next, a 0.2 M aqueous solution of APS radical initiator (0.2 mL, 0.04 mmol, 1 mol% relative to AAm) was added under the protection of N_2_. The first gel network was formed by cooling the mixture to room temperature. The second, PAAm-based network was formed by heating the hydrogel to 65 °C (note: the material was in a gel state at this temperature) for 7 h under an atmosphere of N_2_. The resulting DN hydrogel was then cooled to room temperature. The swelling ratio (*Q*) is defined as: *Q* = (*W*_s_ − *W*_d_)/*W*_d_ where *W*_s_ is the weight of the swollen hydrogel and *W*_d_ is the weight of the dried hydrogel.

## Conflicts of interest

There are no conflicts to declare.

## Supplementary Material

RA-010-D0RA05735E-s001
