# Supplementary material for: Agar-reduced graphene oxide selectively adsorbs organic dyes and strengthens double-network hydrogels
Source: RSC Adv. 2020 Aug 7;10(49):29287–95. doi: 10.1039/d0ra05735e (PMC9055959; doi:10.1039/d0ra05735e)
Supplement: RA-010-D0RA05735E-s001 [file RA-010-D0RA05735E-s001.pdf]

Electronic Supplementary Material (ESI) for RSC Advances.
This journal is © The Royal Society of Chemistry 2020

Supporting Information

Agar-Reduced Graphene Oxide Selectively Adsorbs Organic Dyes and Strengthens Double-Network Hydrogels

Tang Tang,[a] Karel Goossens,[a,d] Sherilyn J. Lu,[a,b] Dongli Meng[a] and Christopher W. Bielawski*[a,b,c]

[a] Center for Multidimensional Carbon Materials (CMCM), Institute for Basic Science (IBS), Ulsan 44919 (Republic of Korea)

[b] Department of Chemistry, Ulsan National Institute of Science and Technology (UNIST), Ulsan 44919 (Republic of Korea)

[c] Department of Energy Engineering, Ulsan National Institute of Science and Technology (UNIST), Ulsan 44919 (Republic of Korea)

[d] Current address: Johnson & Johnson, Janssen R&D, Turnhoutseweg 30, B-2340 Beerse, Belgium.

* Correspondence: bielawski@unist.ac.kr; Tel.: +82-52-217-2952.

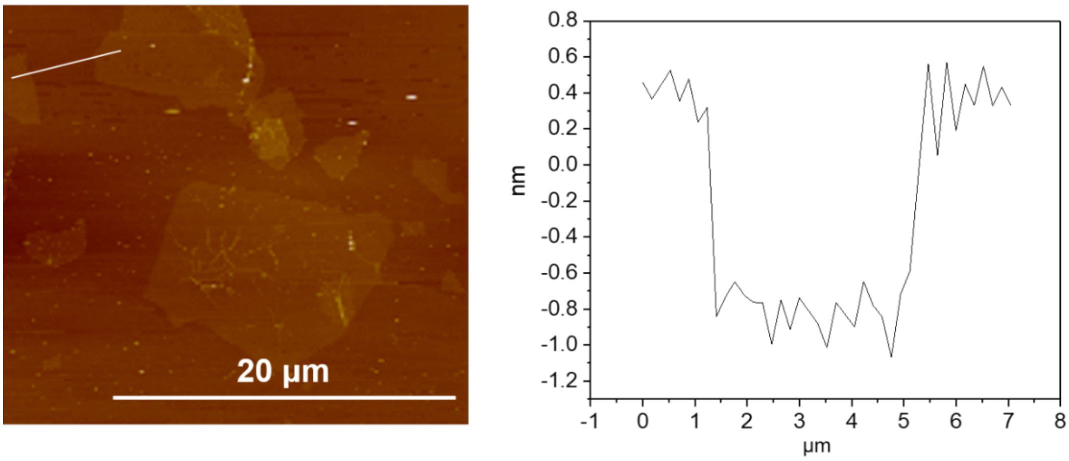

Figure S1. AFM image and profile data recorded for graphene oxide.

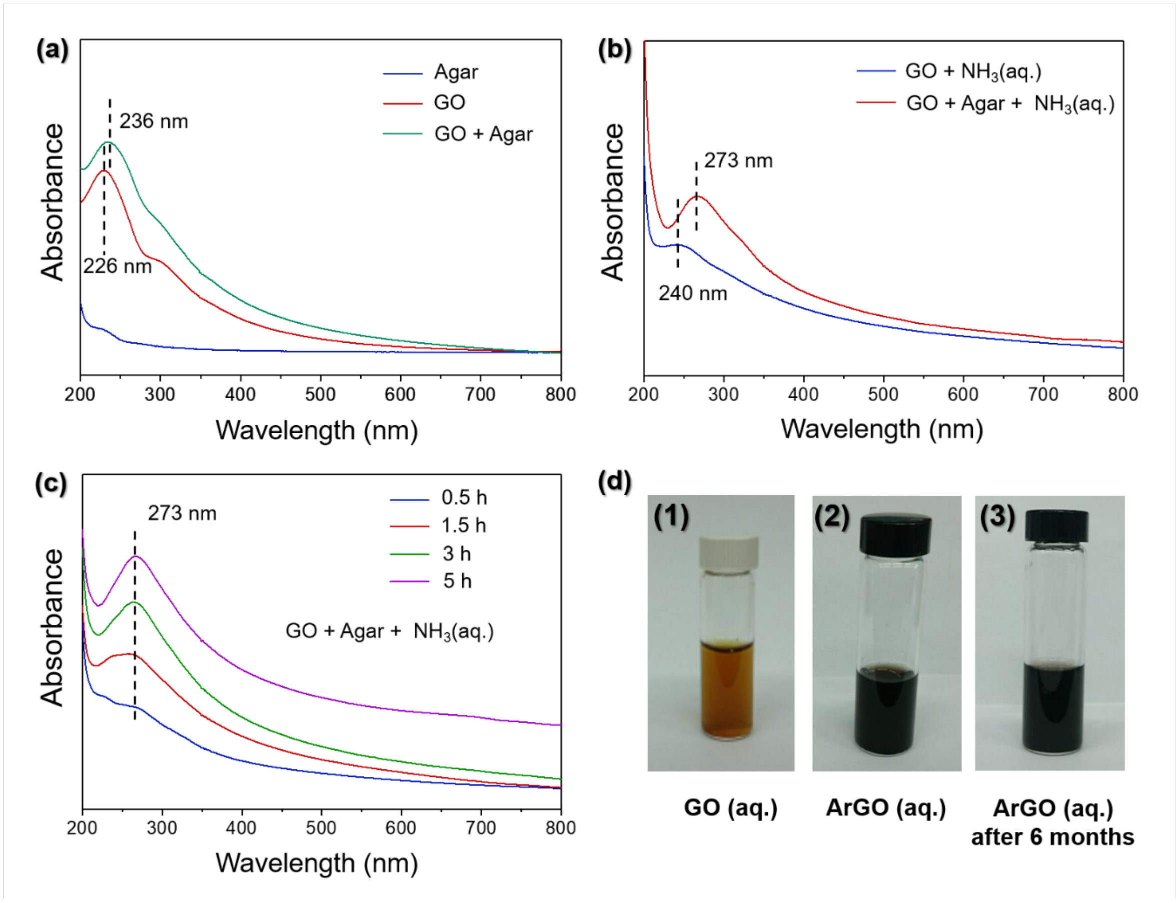

Figure S2. (a) UV–vis absorption spectra that were recorded for a 2.0 mg mL^{-1} aqueous dispersion of agar after being heated to 95 °C for 12 h (blue); a 0.2 mg mL^{-1} aqueous dispersion of GO (red); a 0.2 mg mL^{-1} aqueous dispersion of GO with agar (agar/GO = 10 : 1 w/w) after being heated to 95 °C for 12 h (green). (b) UV–vis absorption spectra that were recorded for a 0.2 mg mL^{-1} aqueous dispersion of GO and NH_3 (aq.) (pH = 8 – 9) after being heated to 95 °C for 3 h (blue); a 0.2 mg mL^{-1} aqueous dispersion of GO, agar (agar/GO = 10 :

1 w/w) and NH_3 (aq.) (pH = 8 – 9) after being heated to 95 °C for 3 h (red). (c) UV–vis absorption spectra that were recorded over time (indicated) for a 0.2 mg mL^{-1} aqueous dispersion of GO, agar (agar/GO = 10 : 1 w/w) and NH_3 (aq.) (pH = 8 – 9) that was kept at 95 °C. (d) Photographs of various suspensions and dispersions (indicated).

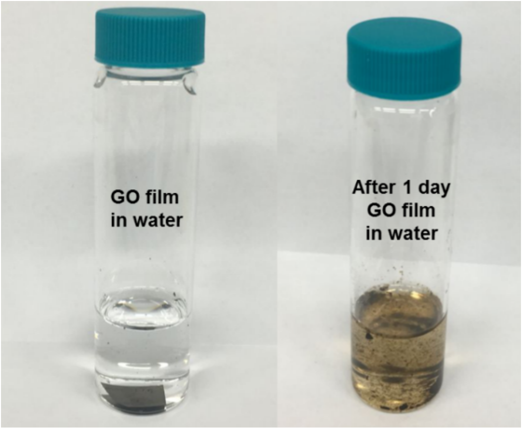

Figure S3. Photographs taken (left) after a film of GO was placed in water and (right) after the mixture was left standing for 1 day.

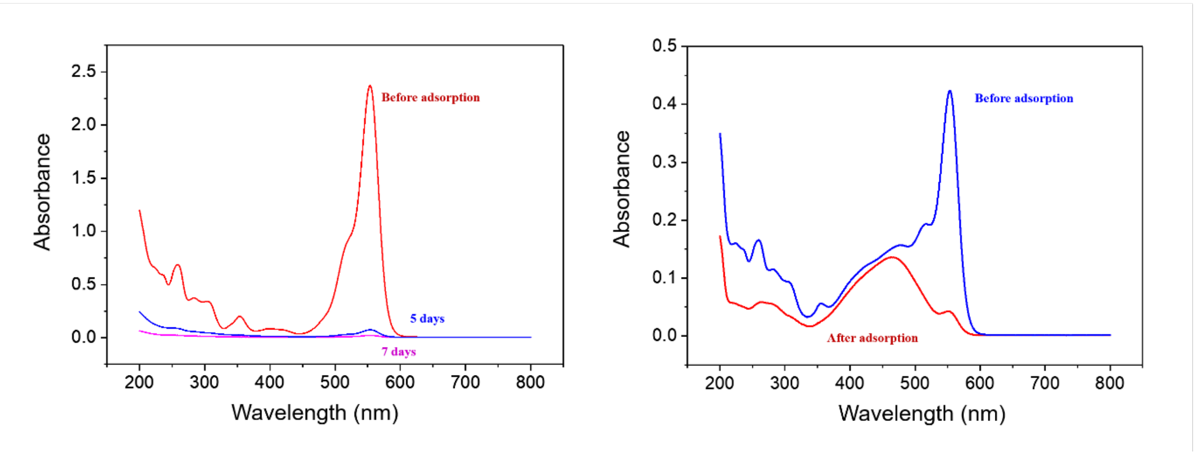

Figure S4. UV-vis spectra. (left) The selective adsorption of RhoB versus MO was demonstrated by soaking ArGO paper (1 mg) in an aqueous mixture of both dyes for 12 h ($[RhoB]_0$ = $[MO]_0$ = 1 mg L^{-1}, 2 mL total volume). (right) The adsorption capacity was assessed by soaking ArGO paper (1 mg) in an aqueous solution of RhoB for 7 days ($[RhoB]_0$ = 20 mg L^{-1}, 2 mL total volume).

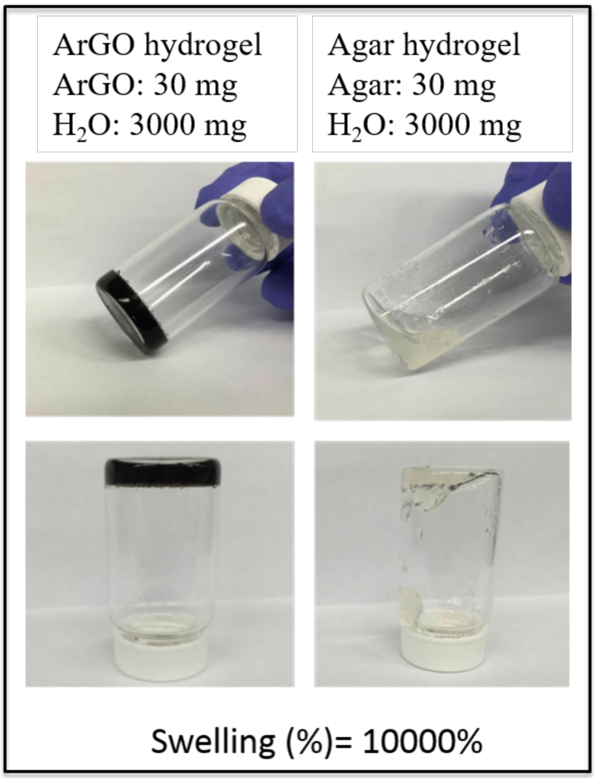

Figure S5. Swelling properties of an ArGO hydrogel and an agar hydrogel (indicated).

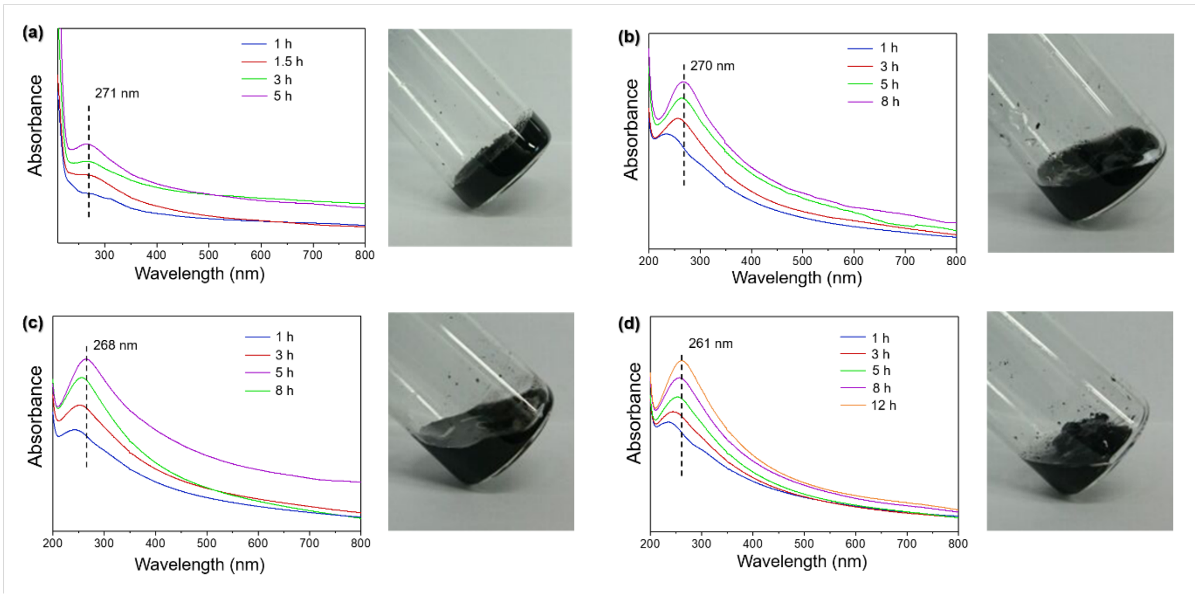

Figure S6. UV-vis absorption spectra that were recorded over time and photographs that were recorded over the sol–gel transition. Conditions: 1.0 mg mL^{-1} aqueous dispersion of GO + NH_3 (aq.) (pH=8-9, mixed with (a) agar (agar/GO = 10 : 1 w/w), (b) agar (agar/GO = 5 : 1 w/w), (c) agar (agar/GO = 2 : 1 w/w) and (d) agar (agar/GO = 1 : 1 w/w). Each mixture was heated to 95 °C for the period indicated in the figure legends. Inset: photographs of the ArGO hydrogels that resulted from the reactions.

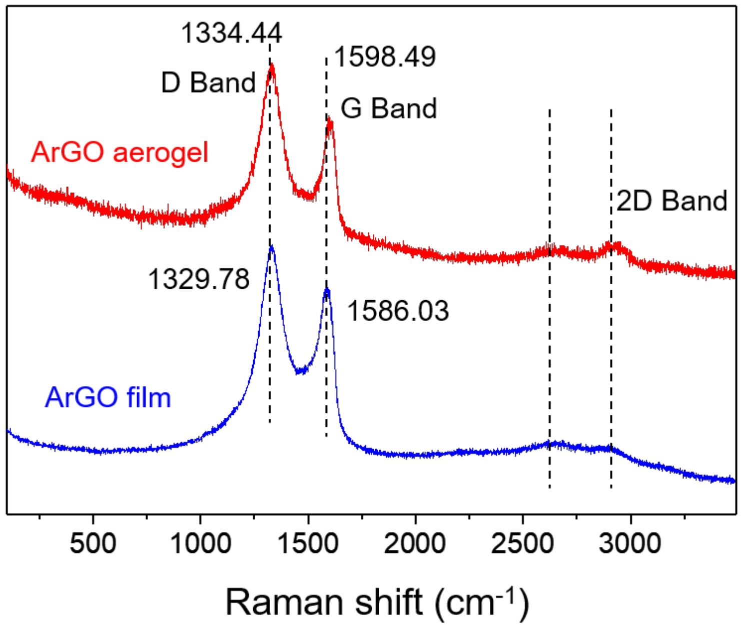

Figure S7. Raman spectra recorded for an ArGO film and an ArGO aerogel (indicated). Conditions: ArGO film, 0.2 mg mL^{-1} aqueous dispersion GO + agar (agar/GO = 10:1 w/w) + NH_3 (aq.) (pH = 8-9), 95 °C for 3 h; ArGO gel, 1.0 mg mL^{-1} aqueous dispersion GO + agar (agar/GO = 10:1 w/w) + NH_3 (aq.) (pH = 8-9), 95 °C for 3 h.

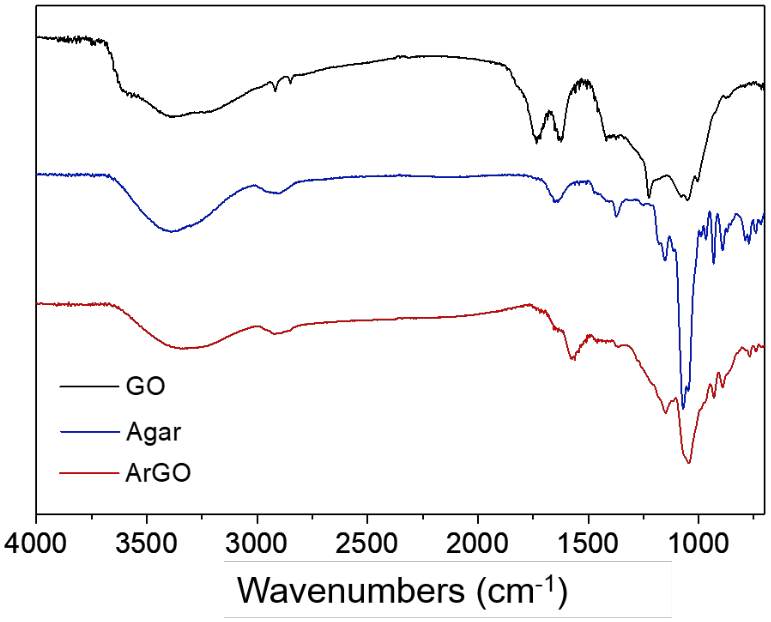

Figure S8. FT-IR spectra recorded for GO, agar and ArGO (indicated). Conditions: 1.0 mg mL^{-1} aqueous dispersion GO + agar (agar/GO = 10:1 w/w) + NH_3 (aq.) (pH = 8-9), 95 °C for 3 h.

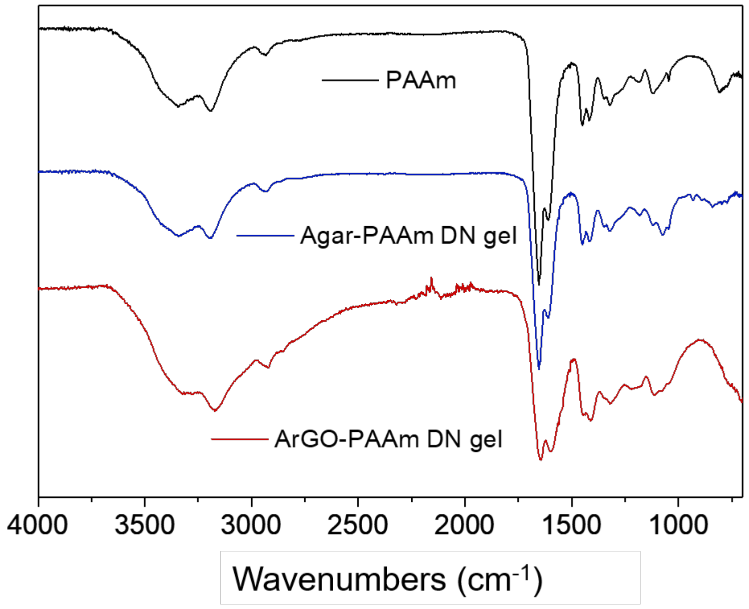

Figure S9. FT-IR spectra recorded for PAAm, Agar-PPAm DN gel and ArGO-PAAm DN aerogel (indicated). The gels were freeze-dried for 2 days and then analyzed. Conditions: PAAm, 0.1 mol% MBAA relative to AAm and 1 mol% APS relative to AAm, polymerization time: 7 h at 65 °C; Agar-PAAm DN hydrogel, Agar/AAm = 1: 9 w/w, 0.1 mol% MBAA relative to AAm and 1 mol% APS relative to AAm, polymerization time: 7 h at 65 °C; ArGO-PAAm DN hydrogel, ArGO/AAm =1: 9 w/w; 0.1 mol% MBAA relative to AAm and 1 mol% APS relative to AAm, polymerization time: 7 h at 65 °C.

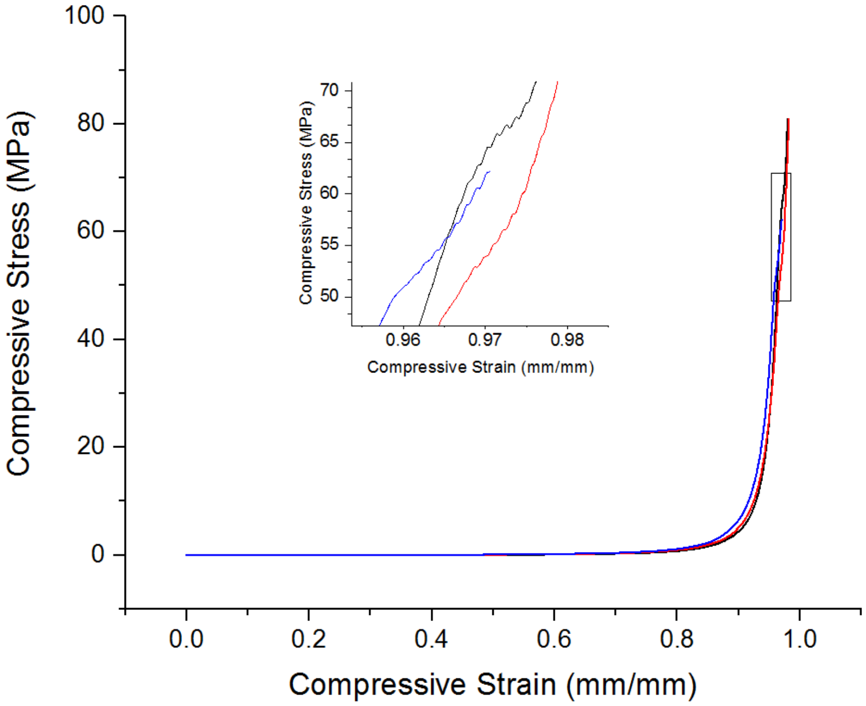

Figure S10. Stress–strain curves that were recorded for an ArGO-PAAm DN hydrogel Conditions: ArGO/AAm = 1 : 9 w/w; 0.1 mol% MBAA relative to AAm and 1 mol% APS relative to AAm; polymerization time: 3.5 h at 65 °C; water content: 83 wt%.

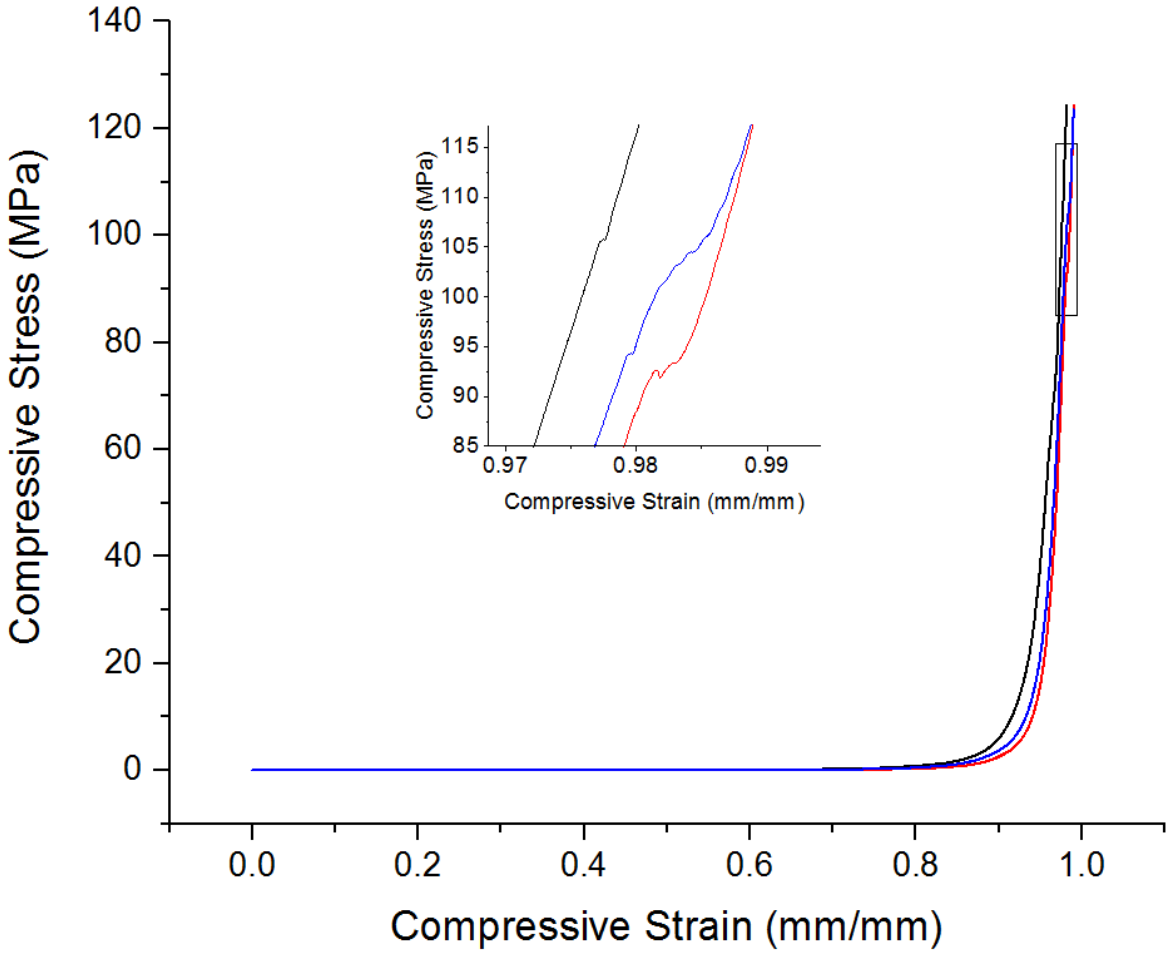

Figure S11. Stress–strain curves that were recorded for an ArGO-PAAm DN hydrogel. Conditions: ArGO/AAm = 1 : 9 w/w, 0.1 mol% MBAA relative to AAm and 1 mol% APS relative to AAm, polymerization time: 7 h at 65 °C; water content: 83 wt%.

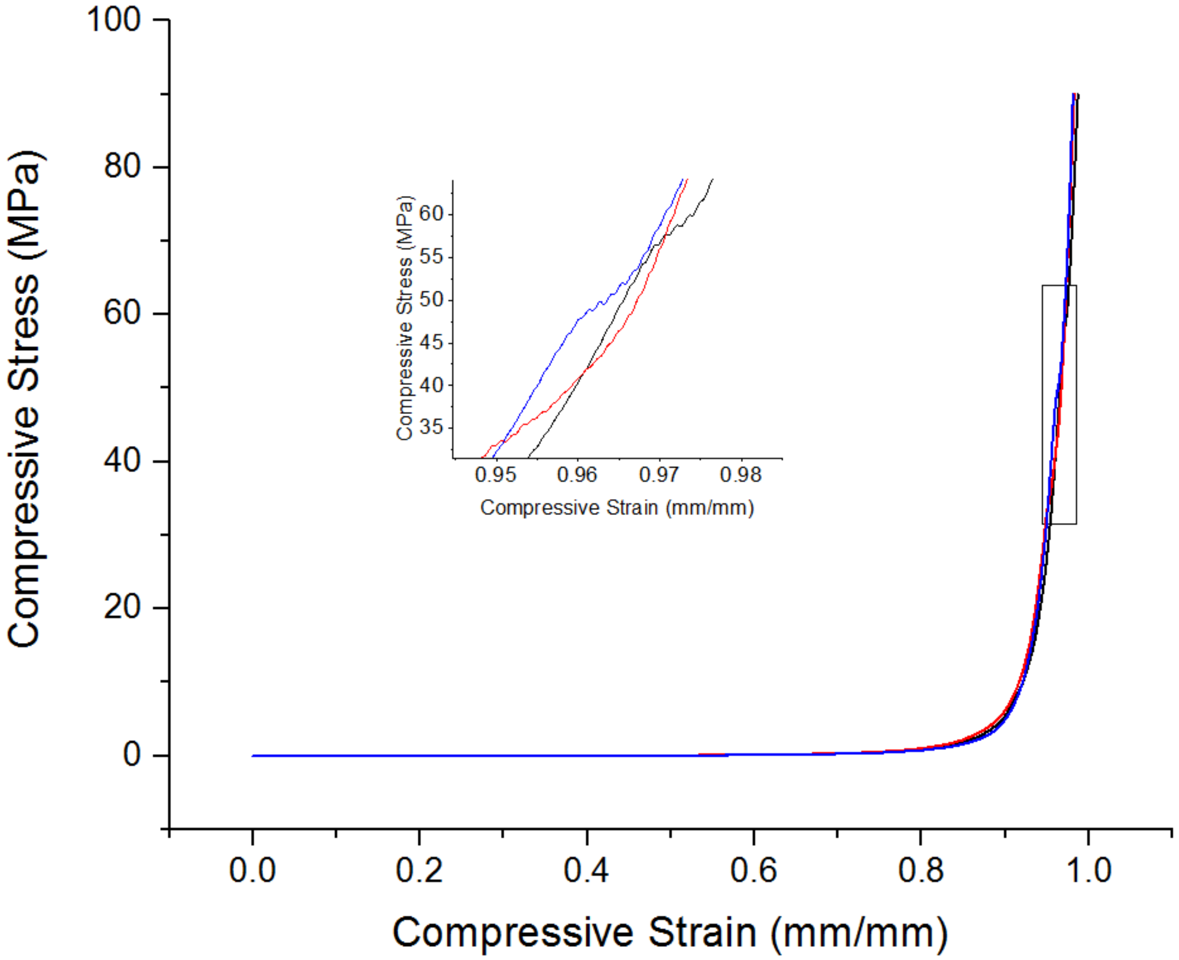

Figure S12. Stress–strain curves that were recorded for an ArGO-PAAm DN hydrogel. Conditions: ArGO/AAm = 1 : 9 w/w; 0.1 mol% MBAA relative to AAm and 1 mol% APS relative to AAm; polymerization time: 12 h at 65 °C; water content: 83 wt%.

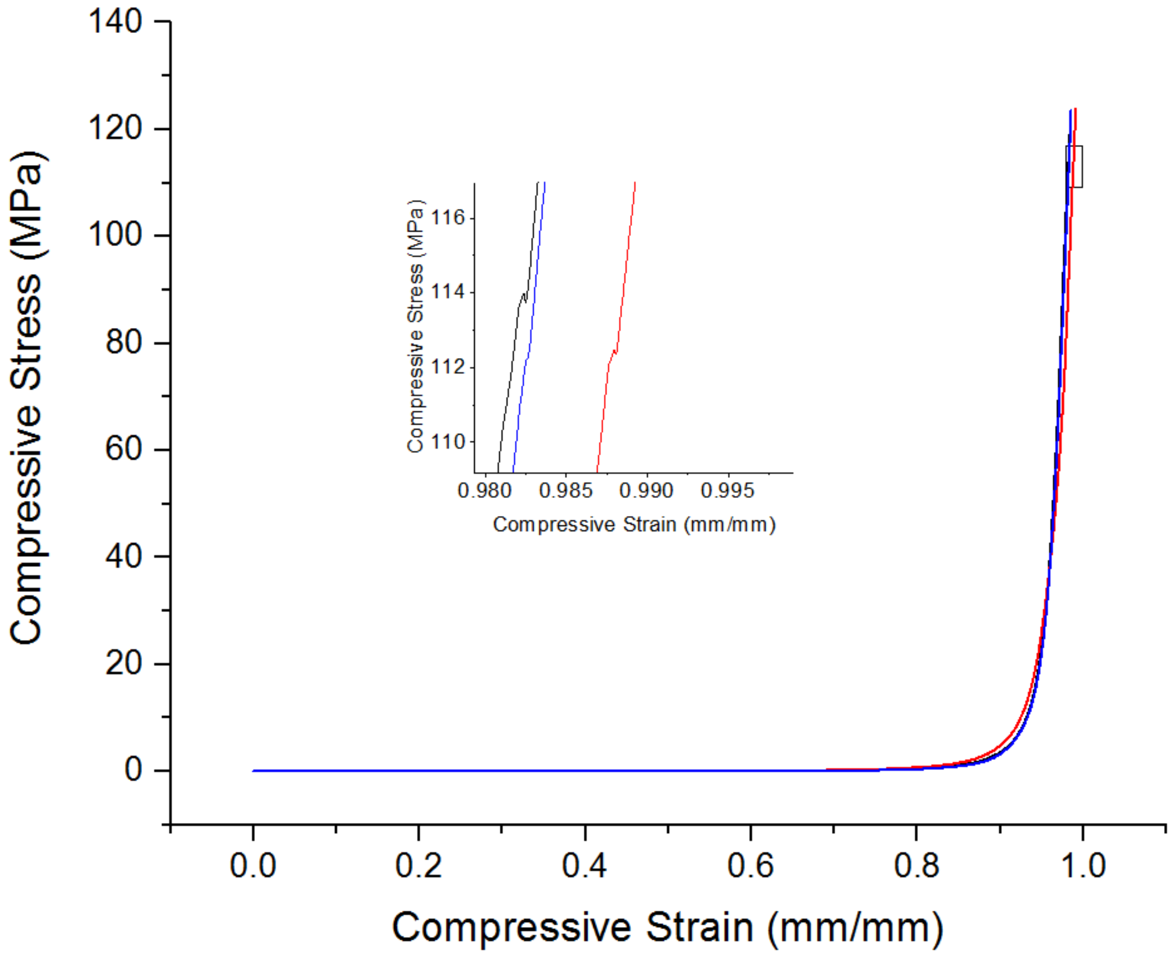

Figure S13. Stress–strain curves that were recorded for an ArGO-PAAm DN hydrogel. Conditions: ArGO/AAm = 1 : 9 w/w, 0.06 mol% MBAA relative to AAm and 1 mol% APS relative to AAm, polymerization time: 7 h at 65 °C; water content: 83 wt%.

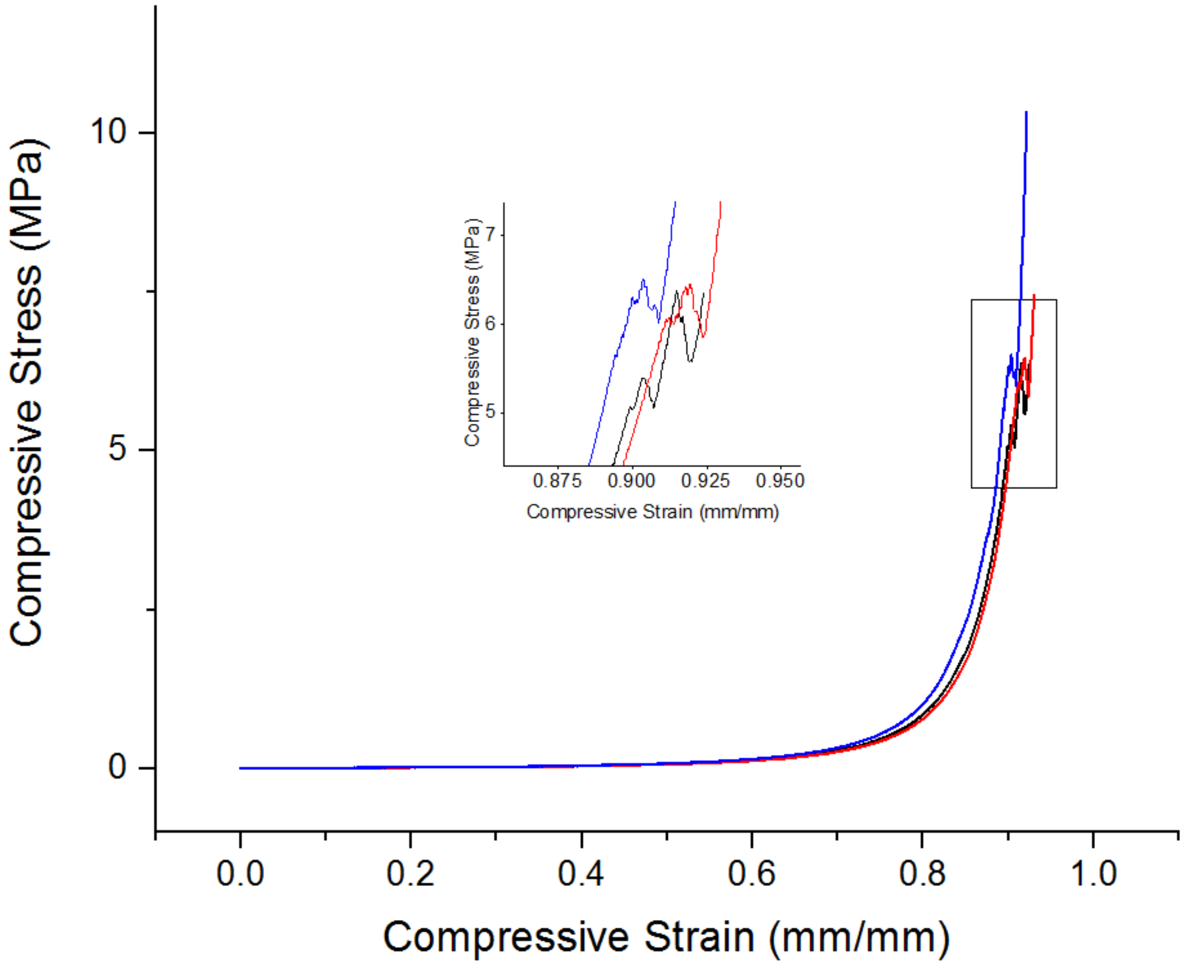

Figure S14. Stress–strain curves that were recorded for an ArGO-PAAm DN hydrogel. Conditions: ArGO/AAm = 1 : 9 w/w, 0.025 mol% MBAA relative to AAm and 1 mol% APS relative to AAm, polymerization time: 7 h at 65 °C; water content: 83 wt%.

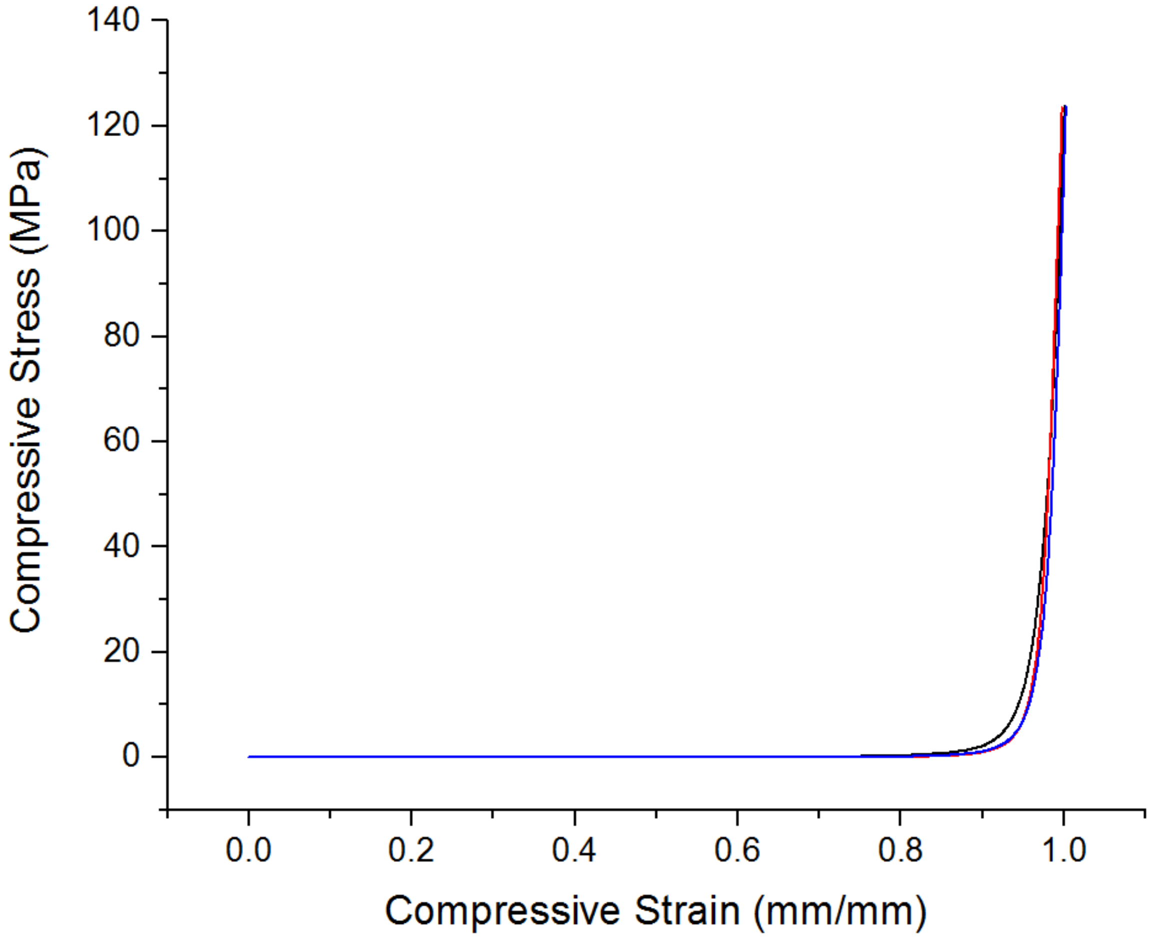

Figure S15. Stress–strain curves that were recorded for an ArGO-PAAm DN hydrogel. Conditions: ArGO/AAm = 1 : 5 w/w, 0.1 mol% MBAA relative to AAm and 1 mol% APS relative to AAm, polymerization time: 7 h at 65 °C; water content: 89 wt%.

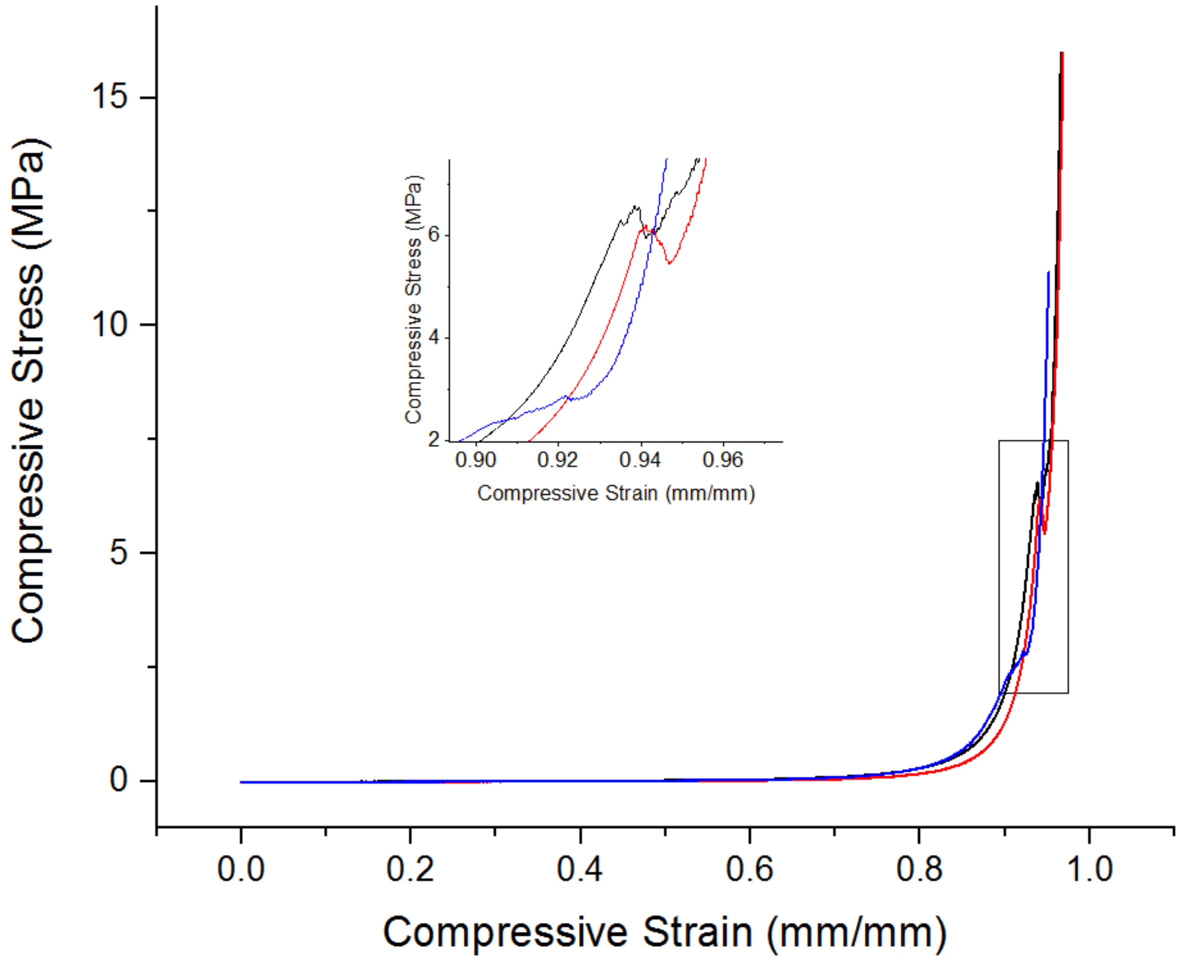

Figure S16. Stress–strain curves that were recorded for an PAAm SN hydrogel. Conditions: 0.1 mol% MBAA relative to AAm and 1 mol% APS relative to AAm, polymerization time: 7 h at 65 °C; water content: 83 wt%.

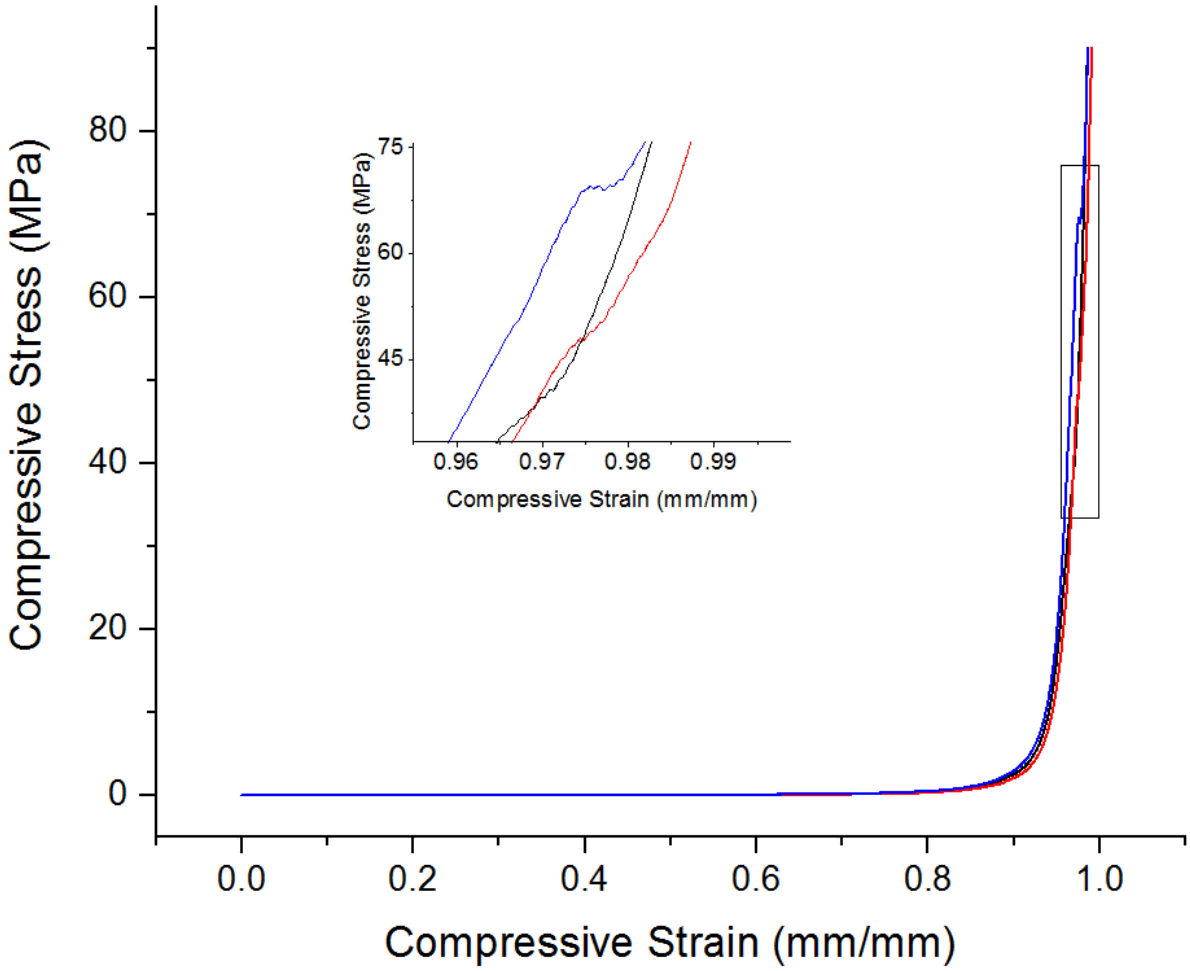

Figure S17. Stress–strain curves that were recorded for an Agar-PAAm DN hydrogel. Conditions: Agar/AAm = 1 : 9 w/w, 0.1 mol% MBAA relative to AAm and 1 mol% APS relative to AAm, polymerization time: 7 h at 65 °C; water content: 83 wt%.
